# Supplementary material for: Molecular Pathogenesis of Colorectal Cancer: Impact of Oncogenic Targets Regulated by Tumor Suppressive miR-139-3p
Source: Int J Mol Sci. 2022 Oct 1;23(19):11616. doi: 10.3390/ijms231911616 (PMC9569794; doi:10.3390/ijms231911616)
Supplement: Supplementary file 1 [file ijms-23-11616-s001.zip › the abbreviations list ijms-1866934.pdf]

the abbreviations list

AGO2	Argonaute2
AKT	RAC-alpha serine/threonine-protein kinase
APC	APC Regulator of WNT Signaling Pathway
CMS	consensus molecular subtypes
CRC	colorectal cancer
ERK	Extracellular Signal-regulated Kinase
FUT1	Fucosyltransferase 1 (H Blood Group)
GAPDH	Glyceraldehyde-3-Phosphate Dehydrogenase
GPRIN2	G Protein Regulated Inducer of Neurite Outgrowth 2
GUSB	Glucuronidase Beta
HE	Hematoxylin and eosin
HK2	hexokinase 2
IRAK1	Interleukin 1 Receptor Associated Kinase 1
KRAS	KRAS Proto-Oncogene
KRT80	Keratin 80
MAPK	Mitogen-activated Protein Kinase
MEK	MAPK/ERK kinase
PCR	Polymerase chain reaction
PI3K	phosphatidylinositol-3 kinase
PIK3CA	Phosphatidylinositol-4,5-Bisphosphate 3-Kinase Catalytic Subunit Alpha
PIP5K1A	Phosphatidylinositol-4-Phosphate 5-Kinase Type 1 Alpha
PRKDC	Protein Kinase, DNA-Activated, Catalytic Subunit
RIP	RNA immunoprecipitation
RISC	RNA-induced silencing complex
SMAD4	SMAD Family Member 4
Ser473	serin 473
TGF-β	Transforming Growth Factor Beta
TP53	Tumor Protein P53
Thr308	threonine 308
UTR	untranslated region
mRNA	messenger RNA
mTOR	mammalian target of rapamycin
mTORC1	mechanistic/mammalian target of rapamycin complex 1

miRNA	microRNA
ncRNA	non-coding RNA
qRT-PCR	quantitative real-time reverse-transcription PCR
siRNA	small interfering RNA
