# Supplementary figures and images for: Molecular Pathogenesis of Colorectal Cancer: Impact of Oncogenic Targets Regulated by Tumor Suppressive miR-139-3p
Source: Int J Mol Sci. 2022 Oct 1;23(19):11616. doi: 10.3390/ijms231911616 (PMC9569794; doi:10.3390/ijms231911616)

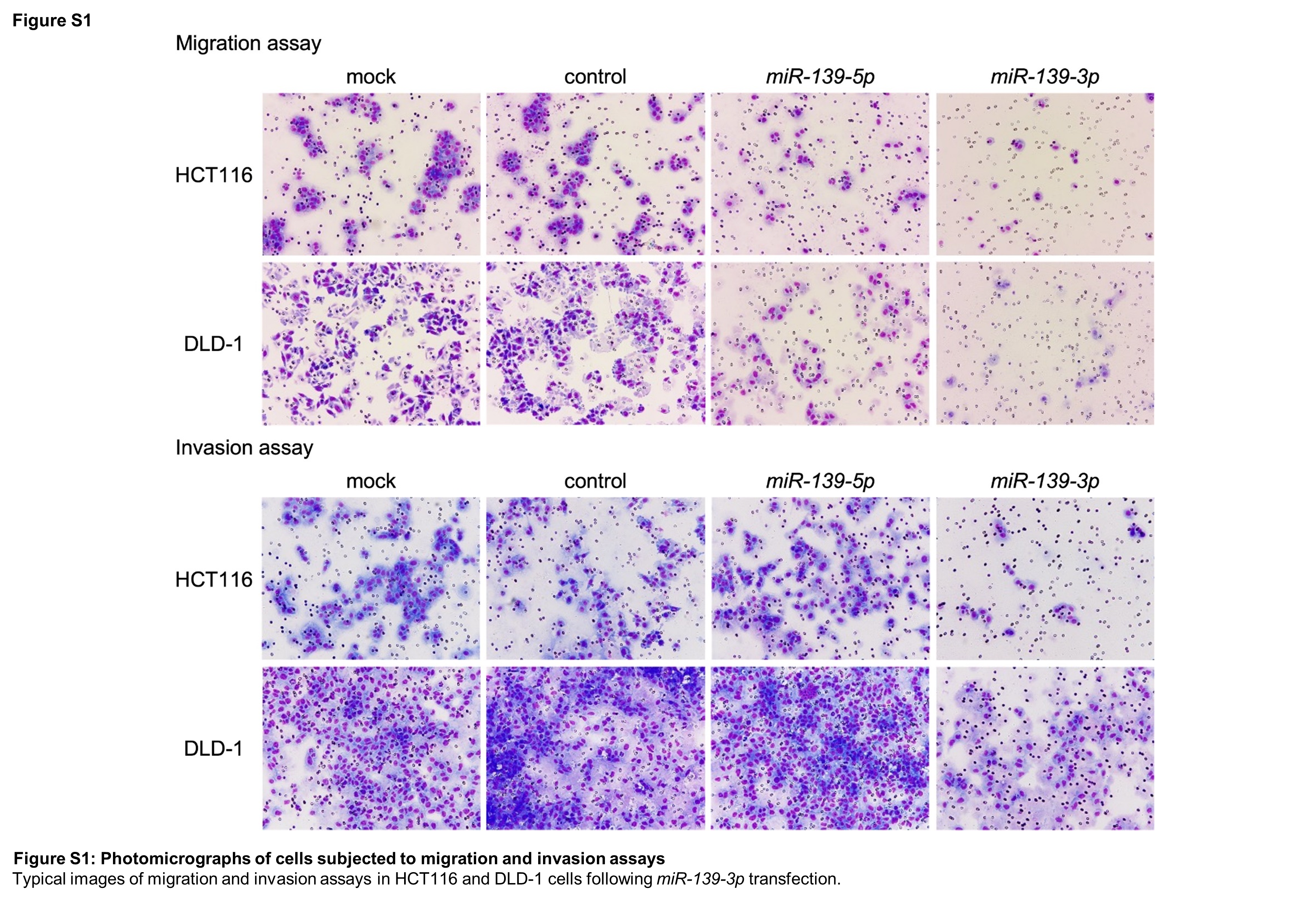

Supplement: Supplementary file 1 [file ijms-23-11616-s001.zip › Proofreading supple-figure ijms-1866934_part1.TIF]

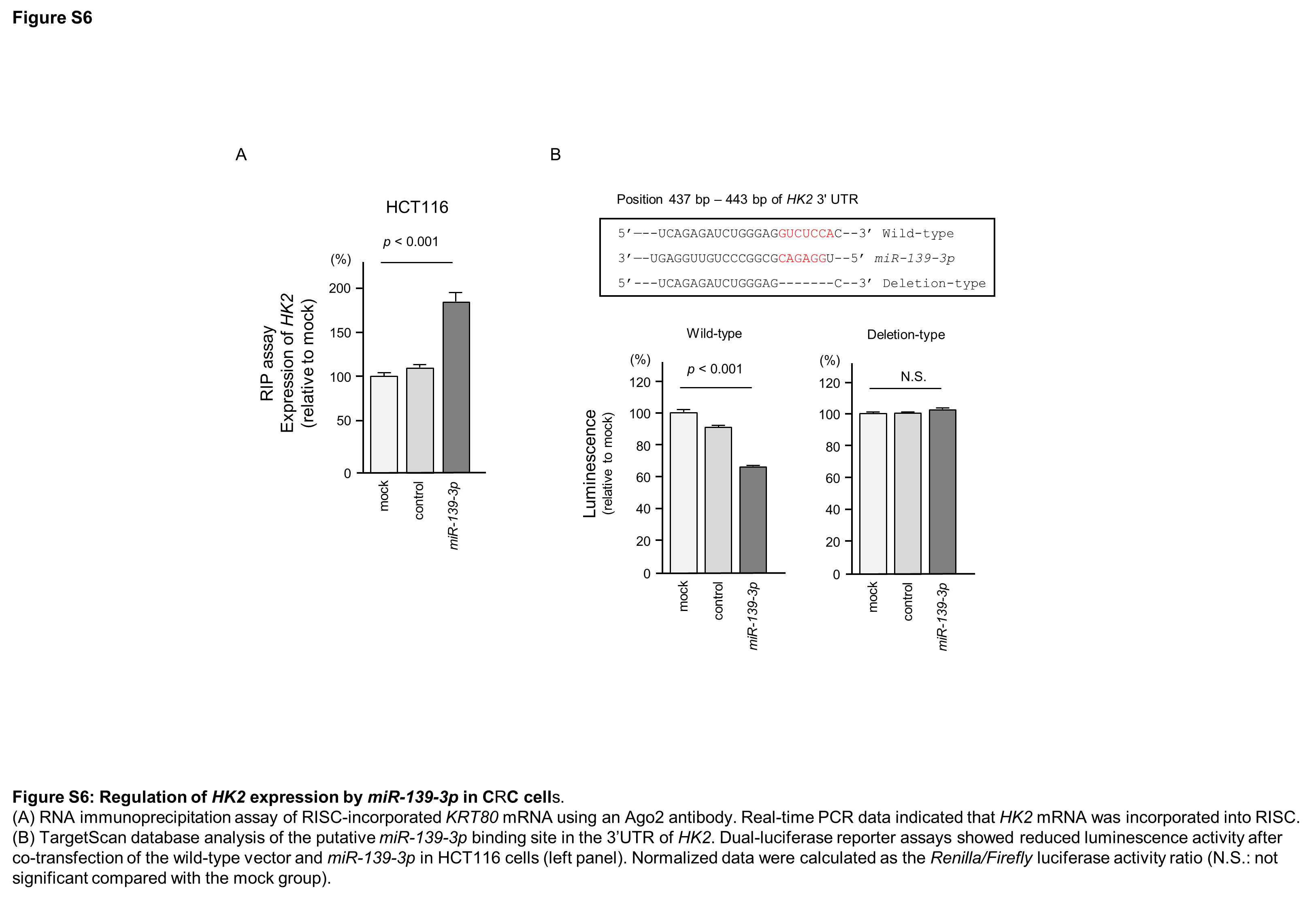

Supplement: Supplementary file 1 [file ijms-23-11616-s001.zip › Proofreading supple-figure ijms-1866934_part10.TIF]

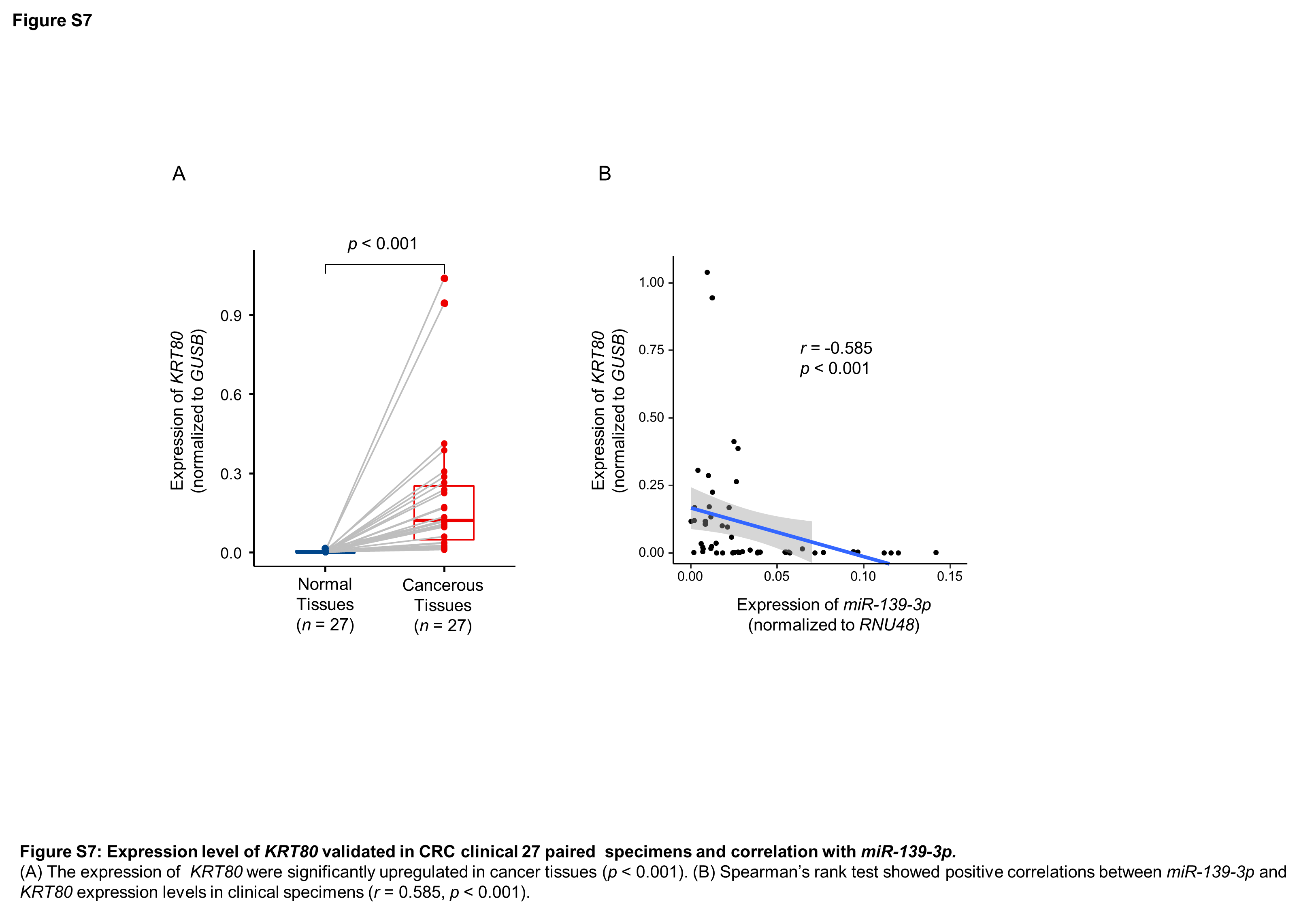

Supplement: Supplementary file 1 [file ijms-23-11616-s001.zip › Proofreading supple-figure ijms-1866934_part11.TIF]

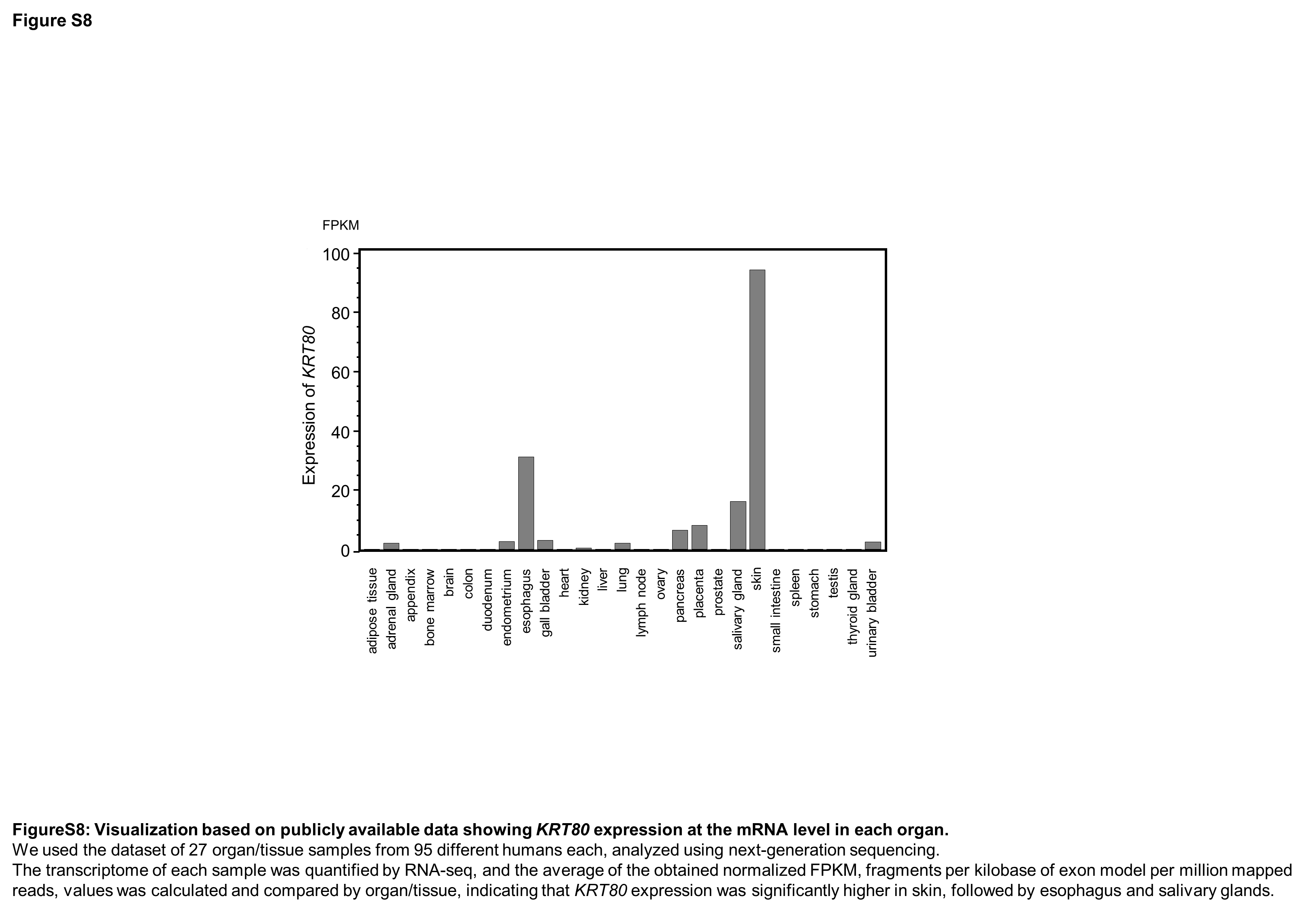

Supplement: Supplementary file 1 [file ijms-23-11616-s001.zip › Proofreading supple-figure ijms-1866934_part12.TIF]

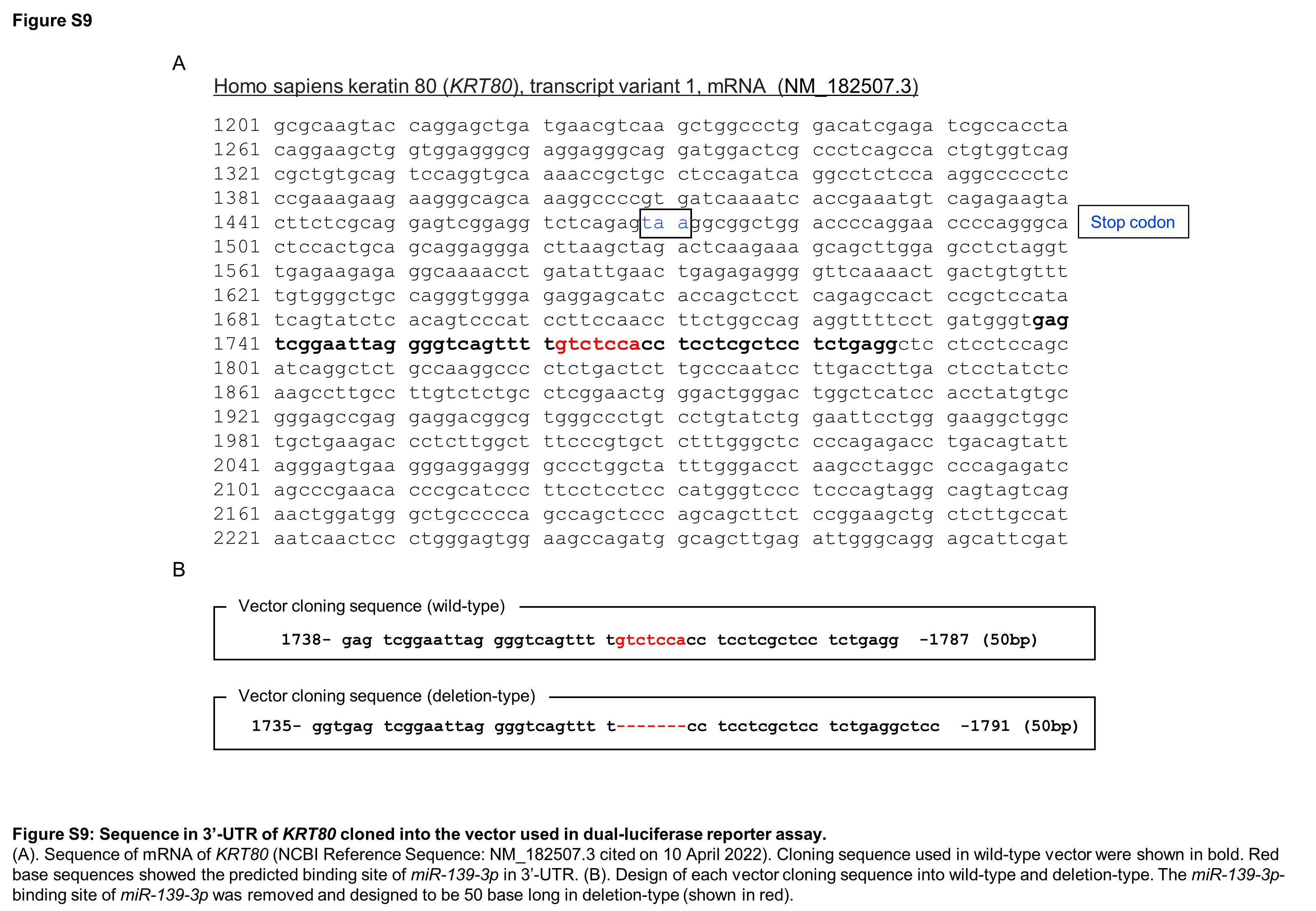

Supplement: Supplementary file 1 [file ijms-23-11616-s001.zip › Proofreading supple-figure ijms-1866934_part13.TIF]

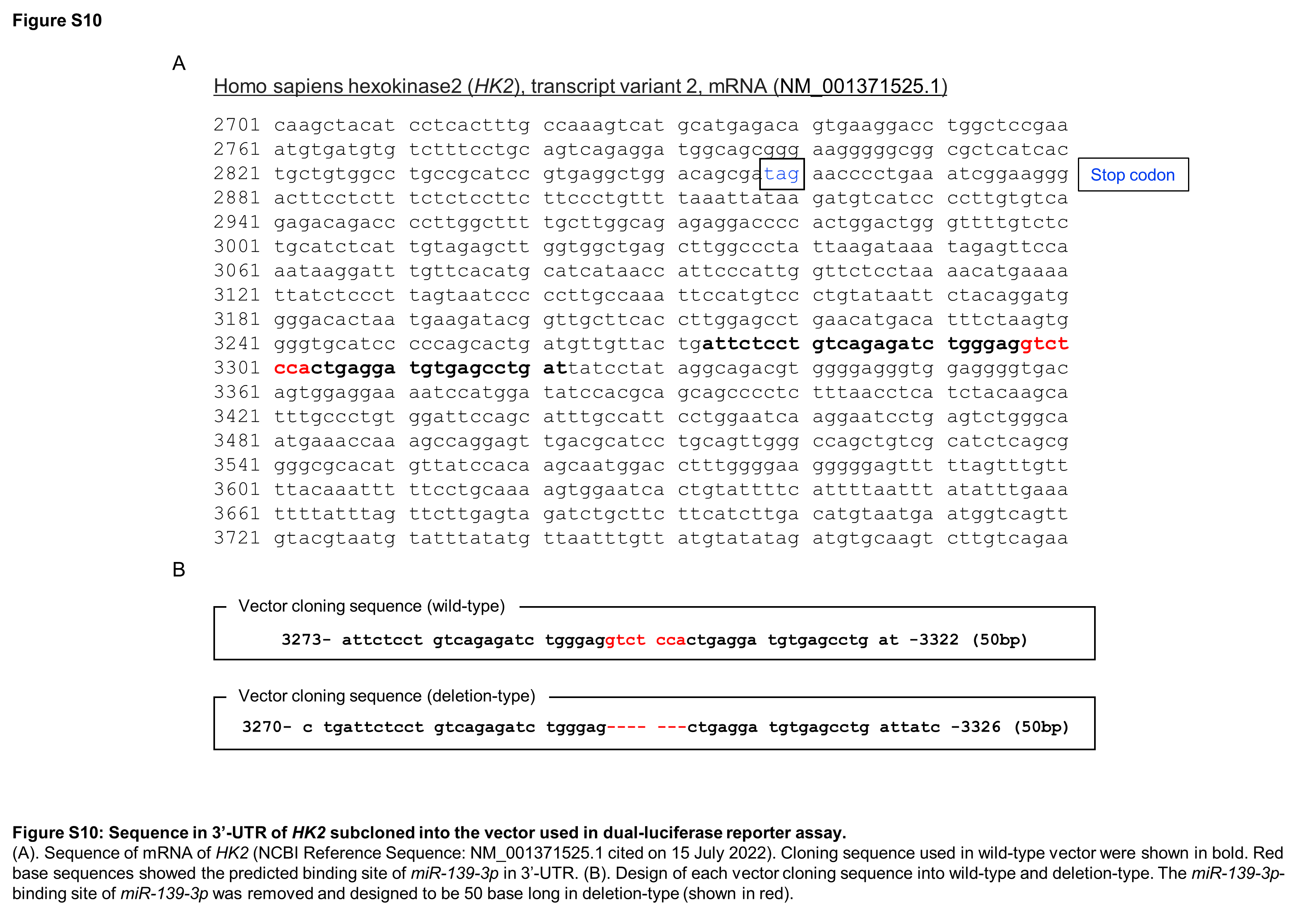

Supplement: Supplementary file 1 [file ijms-23-11616-s001.zip › Proofreading supple-figure ijms-1866934_part14.TIF]

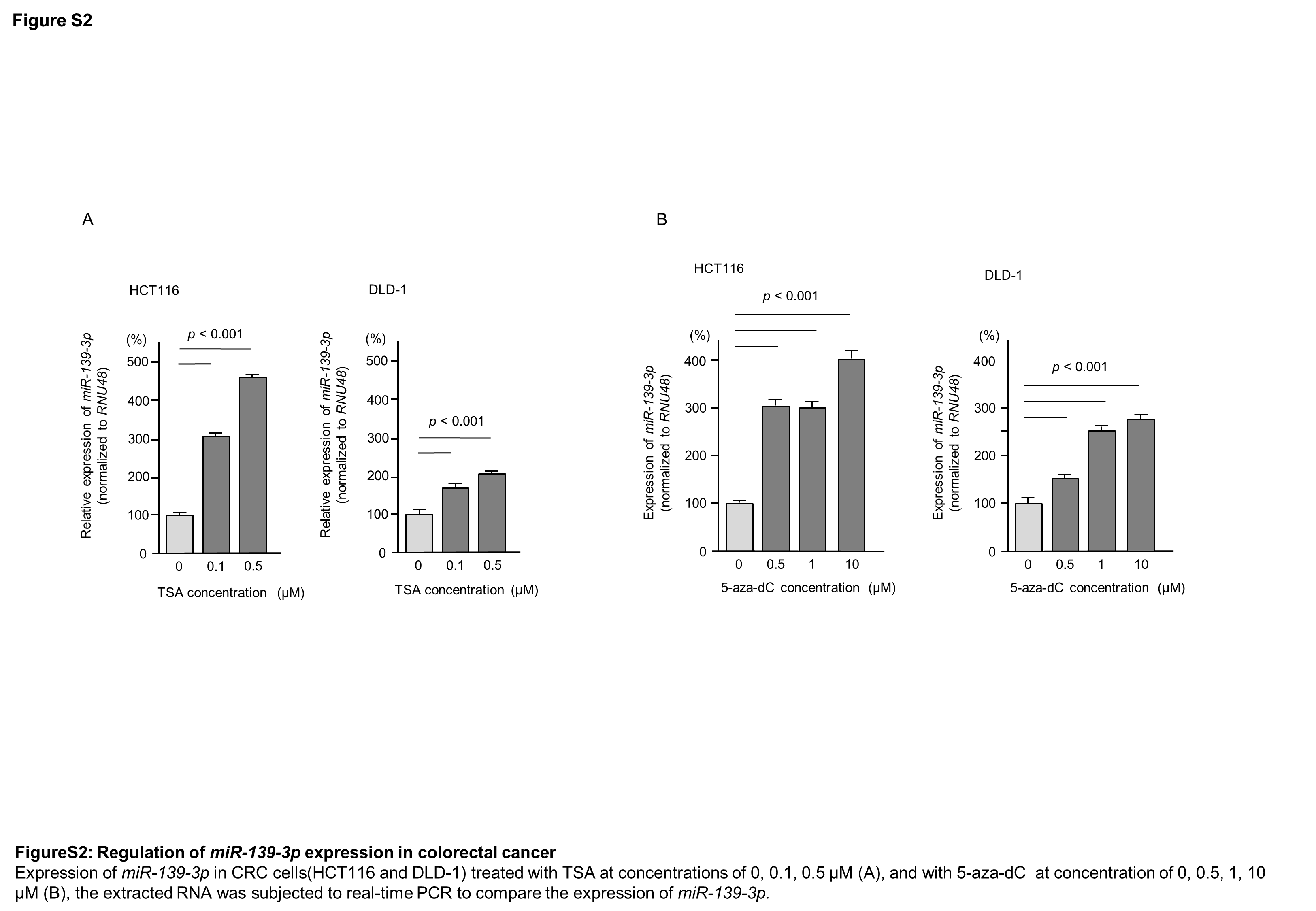

Supplement: Supplementary file 1 [file ijms-23-11616-s001.zip › Proofreading supple-figure ijms-1866934_part2.TIF]

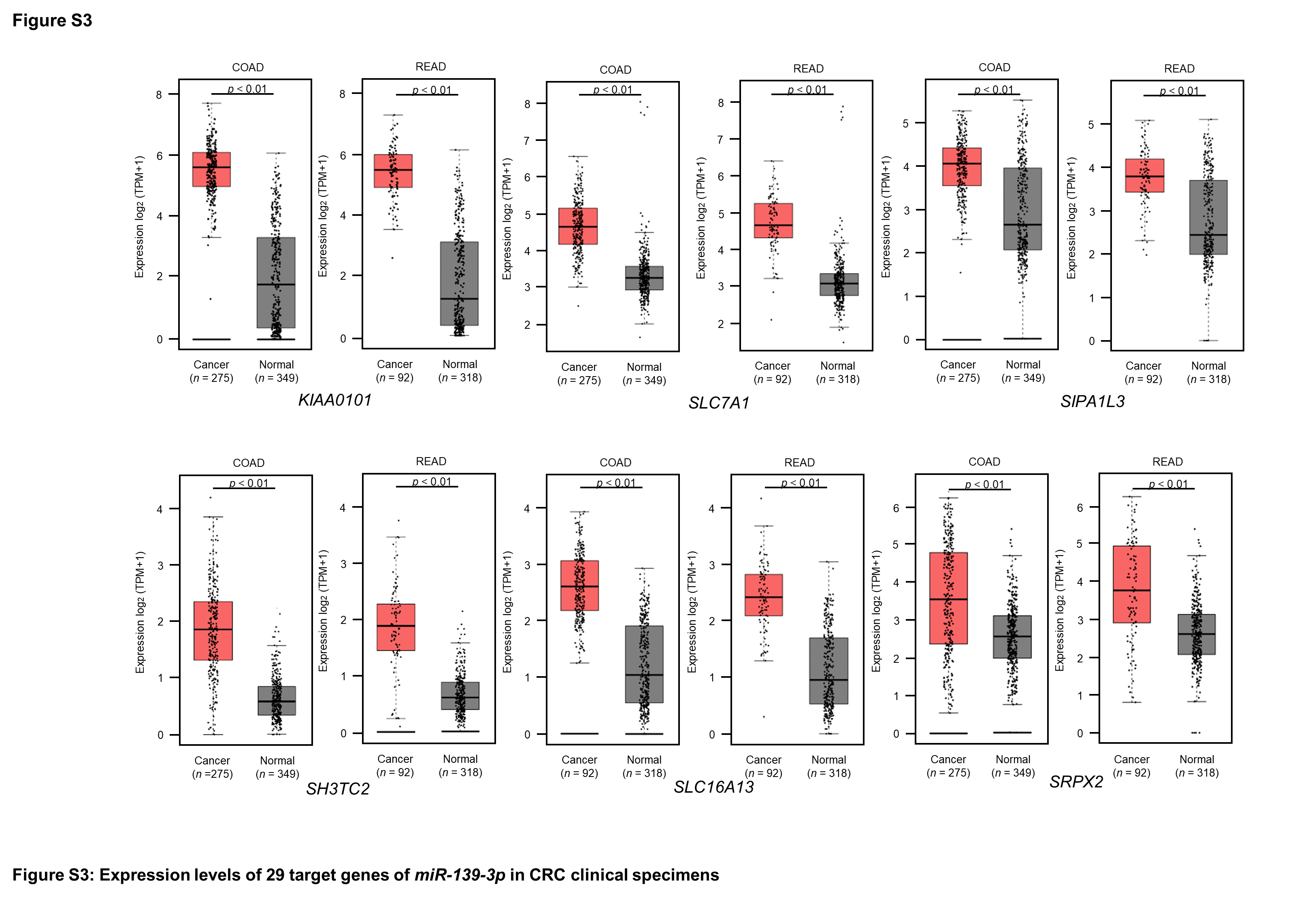

Supplement: Supplementary file 1 [file ijms-23-11616-s001.zip › Proofreading supple-figure ijms-1866934_part3.TIF]

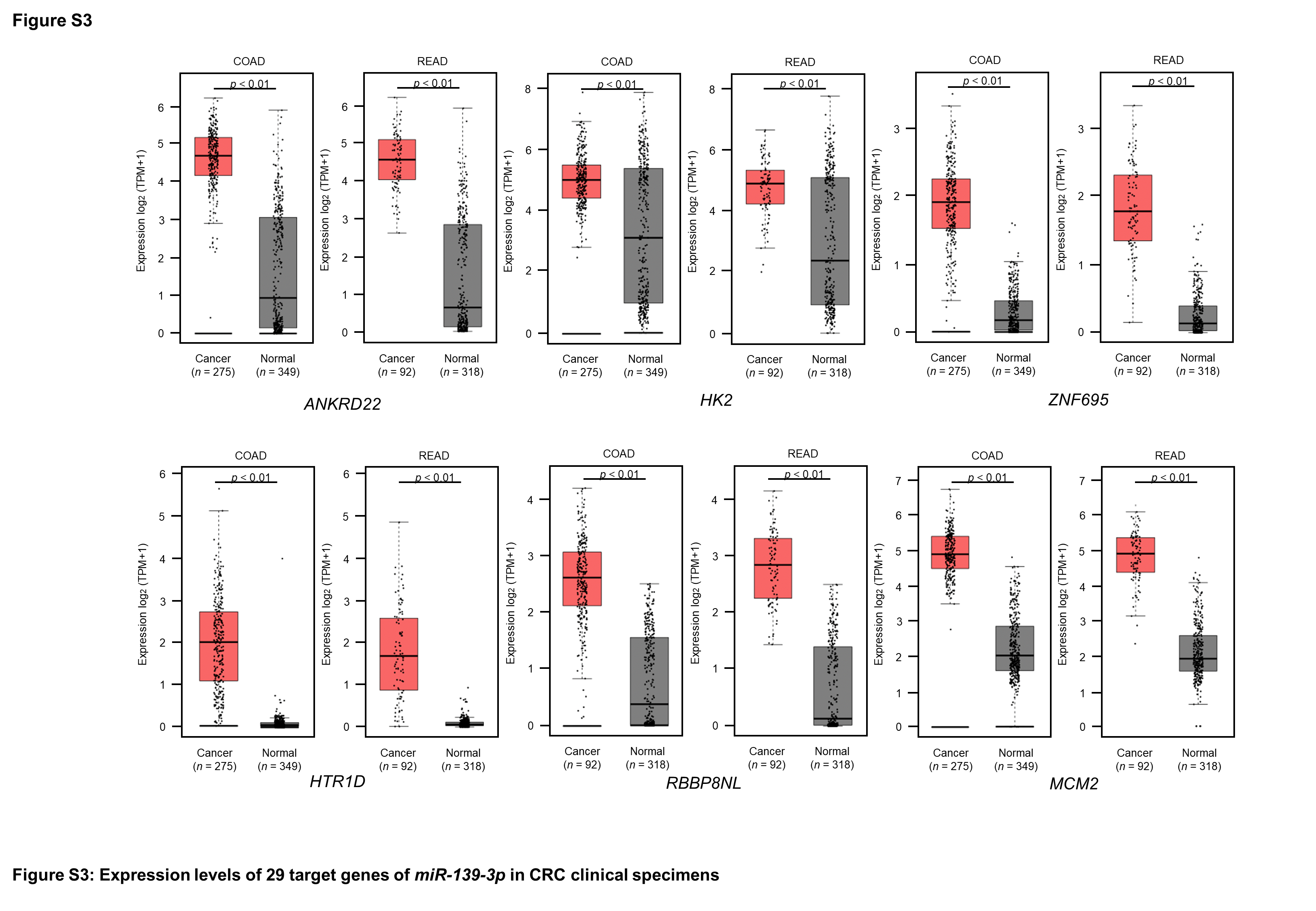

Supplement: Supplementary file 1 [file ijms-23-11616-s001.zip › Proofreading supple-figure ijms-1866934_part4.TIF]

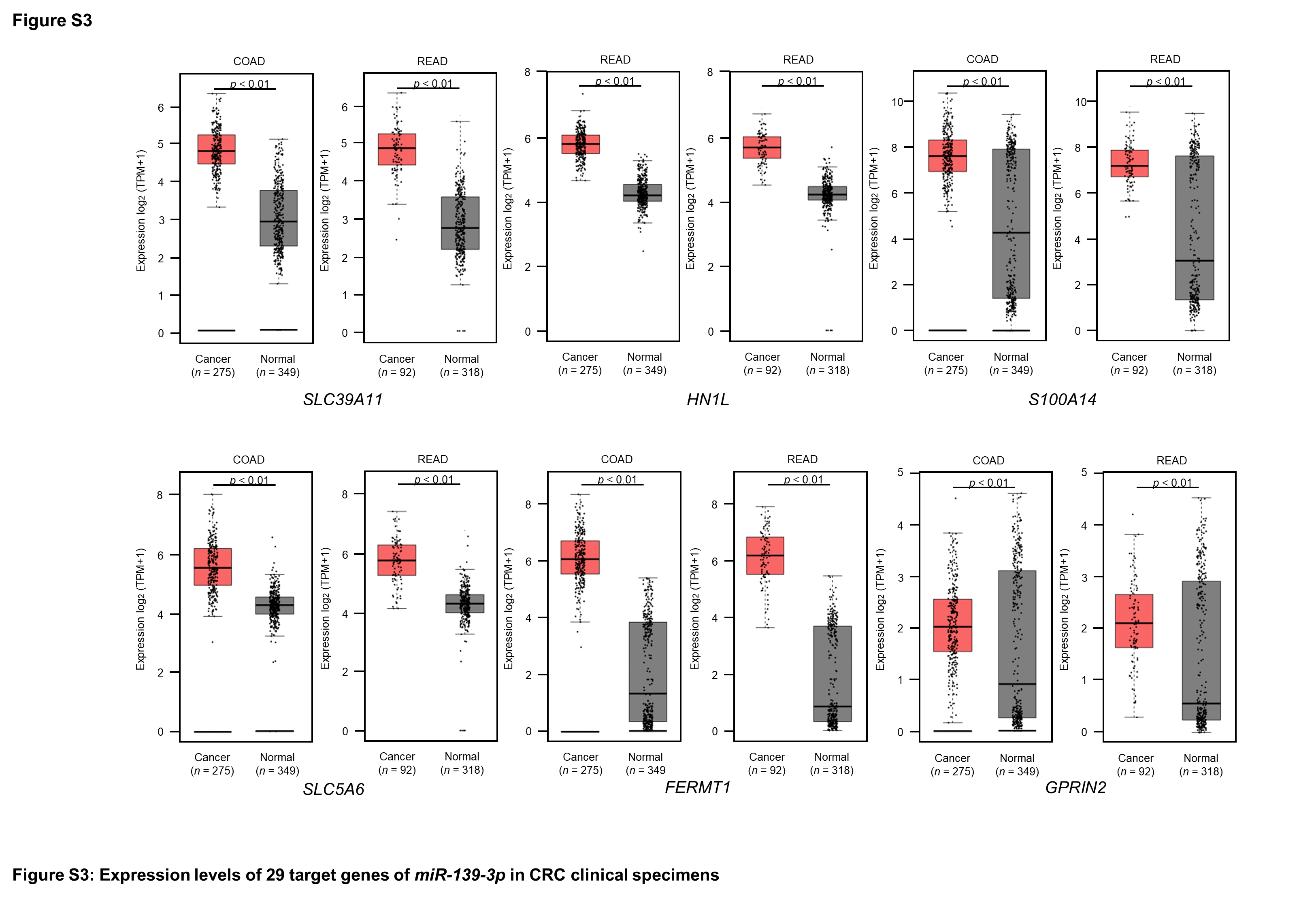

Supplement: Supplementary file 1 [file ijms-23-11616-s001.zip › Proofreading supple-figure ijms-1866934_part5.TIF]

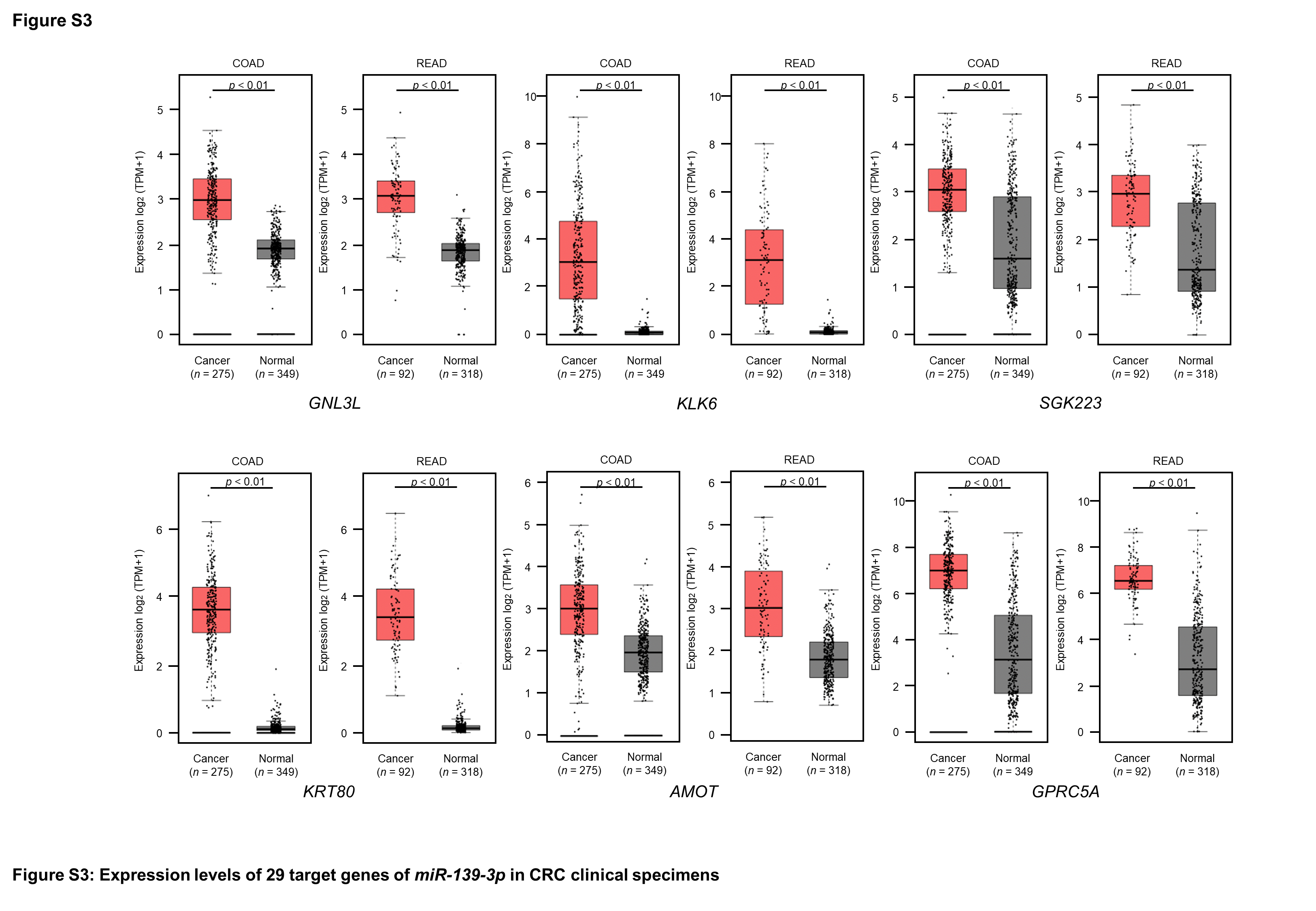

Supplement: Supplementary file 1 [file ijms-23-11616-s001.zip › Proofreading supple-figure ijms-1866934_part6.TIF]

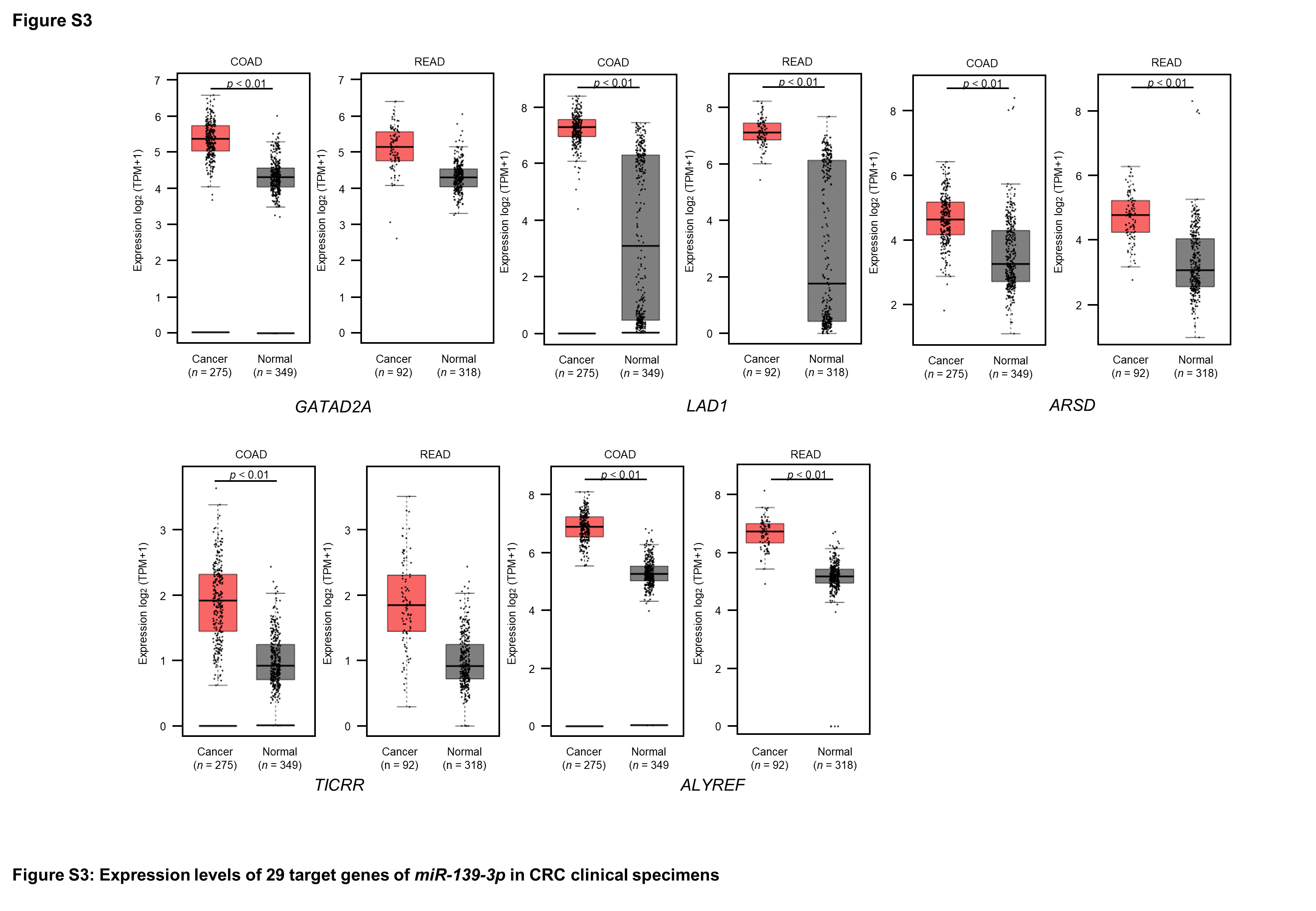

Supplement: Supplementary file 1 [file ijms-23-11616-s001.zip › Proofreading supple-figure ijms-1866934_part7.TIF]

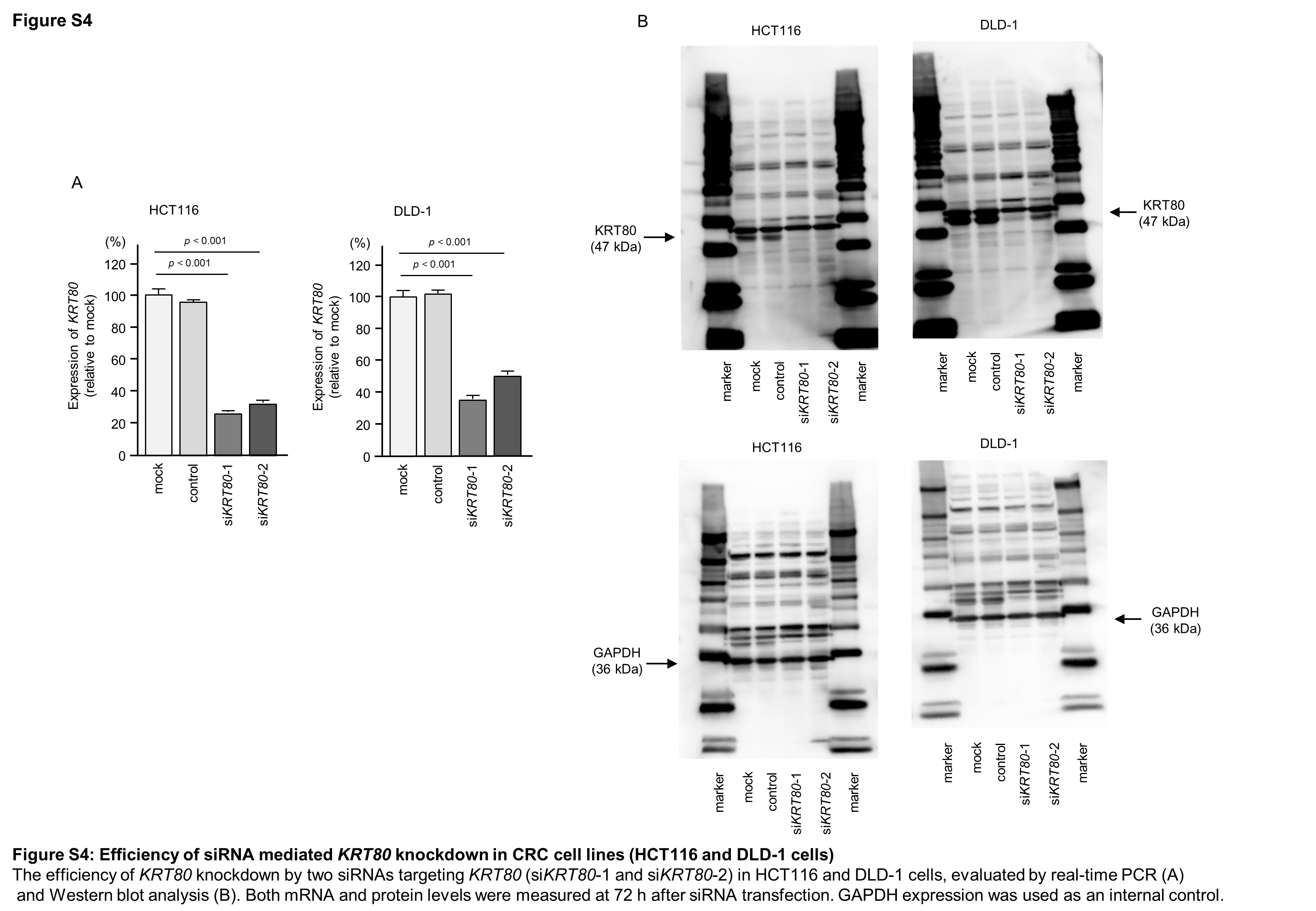

Supplement: Supplementary file 1 [file ijms-23-11616-s001.zip › Proofreading supple-figure ijms-1866934_part8.TIF]

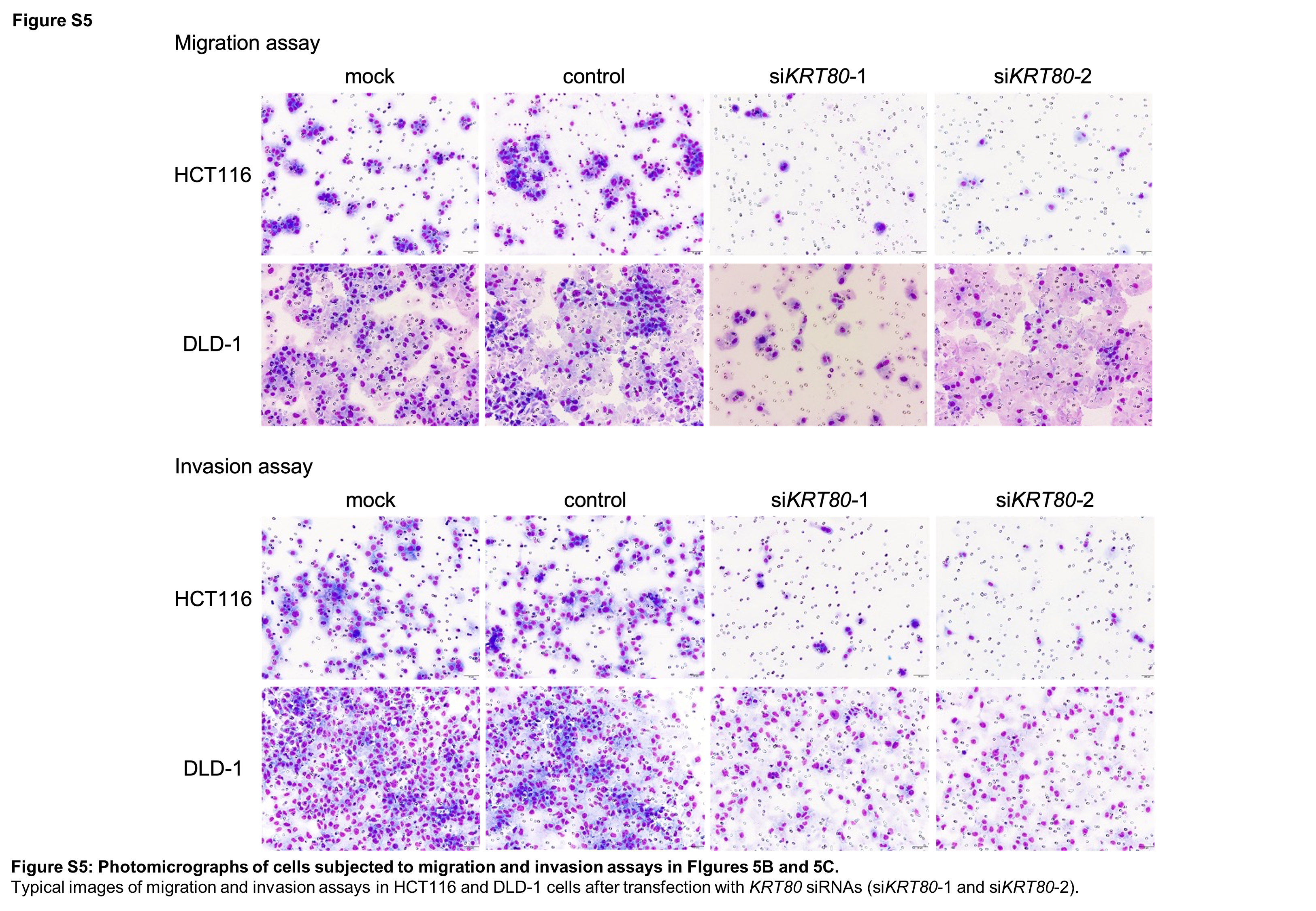

Supplement: Supplementary file 1 [file ijms-23-11616-s001.zip › Proofreading supple-figure ijms-1866934_part9.TIF]
